# Acupuncture Effect on Reaction-Time Changes in Parkinson’s Disease Patients—Case Study Series

**DOI:** 10.3390/jcm13185642

**Published:** 2024-09-23

**Authors:** Catarina Ramos Pereira, Henry J. Greten, Rubim Santos, Ana Mafalda Reis, Bruno Ramos, Maria João Santos, Jorge Machado, Maria Begoña Criado

**Affiliations:** 1 ICBAS—Abel Salazar Institute for Biomedical Sciences, University of Porto, 4099-002 Porto, Portugaljmachado@icbas.up.pt (J.M.); 2 Piaget Institute, Vila Nova de Gaia, 4099-002 Porto, Portugal; 3 CBSIn—Center of Biosciences in Integrative Health, 4099-002 Porto, Portugal; 4 Academia de Saúde C+, 4099-002 Porto, Portugal; 5 DGTCM—German Society of Traditional Chinese Medicine, 69126 Heidelberg, Germany; 6 HSCM—Heidelberg School of Chinese Medicine, 69126 Heidelberg, Germany; 7 ESS, Polytechnic of Porto, 4099-002 Porto, Portugal; rss@ess.ipp.pt; 8 Hospital Pedro Hispano, 4464-513 Matosinhos, Portugal; 9 1H-TOXRUN—Toxicology Research Unit, University Institute of Health Sciences, CESPU, CRL, 4585-116 Gandra, Portugal

**Keywords:** acupuncture, traditional chinese medicine, heidelberg model, parkinson’s disease, reaction time, biopac system

## Abstract

**Background:** Parkinson’s Disease (PD) is a progressive neurodegenerative condition associated with deficit in reaction time which can lead to falls, resulting in limited independence, diminished quality of life, heightened rates of institutionalization and increased healthcare costs. We aimed to examine the effects of an acupuncture protocol in motor time response after an auditory stimulus. **Methods**: This study employed a case series design. Reaction time to exposed rhythmic and random auditory stimuli outcomes were evaluated at six different moments over a month-long acupuncture treatment protocol using the MP 36 system from Biopac Systems. **Results**: We observed a tendency to have more pronounced improvements in the time response in the more affected side of the body compared with the contralateral one. Patients tended to show better values of response to random auditory stimuli compared to rhythmic auditory ones. We also observed a tendency to obtain better results when considering the accumulative effects of the acupuncture protocol. **Conclusions**: Our findings indicated a possible role of reaction time as a sensitive and useful tool for motor function assessment in PD patients. Also, from our results, we concluded that the acupuncture protocol used may lead to an improvement in efficacy of motor response after aleatory and rhythmic stimulus; we also found a tendency for a higher efficacy of acupuncture in random stimuli responses in the first stages of the disease. However, further in-depth research, including a statistical evaluation with a larger participant pool, is necessary to validate and confirm these promising results.

## 1. Introduction

Parkinson’s disease (PD) is a progressive neurodegenerative condition associated with the deposition of aggregated α-synuclein [1] that substantially diminishes quality of life, leading to heightened rates of institutionalization and increased healthcare costs [2]. In recent years, its prevalence has increased globally, making this disease more relevant [3].

Rest tremor, bradykinesia, rigidity and loss of postural reflexes are well-known major signs of PD [4]. As there is no specific examination that validates the presence of the disease, the diagnose is based on the observation of these symptoms by a clinician, which can create difficulties in the examination of early-stage PD patients, due to possible overlaps in the clinical manifestation of the patients with atypical Parkinsonism’s. This leads to a high percentage of underdiagnosed cases or wrong diagnoses [5,6]. However, the disease can manifest many other motor and non-motor symptoms.

PD is characterized by a disruption in the motor circuit of the basal ganglia caused by the loss of dopaminergic neurons in the nigrostriatal pathway. This contributes to the emergence of symptoms such as gait deficit, characterized by reduced postural adjustments and postural instability, typically resulting in falls. Postural instability has been correlated with slower information-processing speed.

In fact, balance information provided by the peripheral sensory organs (eyes, muscles, and joints) and the two sides of the vestibular system, is sent to the brain stem. There, it is sorted out and integrated with learned information contributed by the cerebellum (the coordination center of the brain) and the cerebral cortex (the thinking and memory center). The cerebellum provides information about automatic movements that have been learned through repeated exposure to certain motions. This entire peripheral and central sensory process will always involve an enormous amount of information to make constant postural adjustments in fractions of milliseconds. Consequently, a simple reaction-time task can be used to measure the speed of information processing at the correct time, in real time [7].

Reaction time involves various physiological structures and neural pathways that work together to process sensory stimuli and generate a motor response, including sensory cortex, primary motor cortex, pre-motor cortex and parietal cortex, basal ganglia, thalamus, cerebellum, corticospinal tract, spinal cord, and motor neurons and the peripheral nervous system. It is known that in Parkinson’s disease there is a degeneration of dopaminergic neurons in the substantia nigra that affects the basal ganglia, which implies that reaction time can be negatively impacted in this condition.

PD is found to cause a consistent deficit in simple reaction time (RT) [8,9], with increased time response, compared to healthy controls [10]. This is one of the main causes of postural instability, which leads to reduced independence and frequently results in falls in these patients. [11,12]. PD patients have reduced ability to adapt their gait to unexpected targets or obstacles, and exhibit poorer stepping responses, particularly in a test condition involving conflict resolution [13].

Current pharmacotherapy has limited efficacy and intolerable side effects in late-stage PD [14]. Many patients develop motor complications that are uncontrolled by levodopa or other drug dose adjustment. More than that, several sources of evidence showed that, after 3–5 years of prolonged used, levodopa causes dyskinesias. So, we can consider that there is no current effective medical or chirurgical treatment that can prevent or reverse the progression of PD [15].

Presently, the treatment for Parkinson’s disease consists of (a) medication therapy (levodopa, dopamine agonists, MAO-B inhibitors, COMT inhibitors, anticholinergics, and amantadine), (b) surgical therapy, namely Deep Brain Stimulation (DBS), and (c) physical therapy, including Physiotherapy, Occupational Therapy, and Speech Therapy. New treatments are also being studied, such as gene therapy, stem cell therapies, and α-synuclein inhibitors. 

Because conventional treatment does not give an effective response, some patients search for complementary treatments, such as acupuncture. 

Acupuncture has been used to address the complicated symptoms of PD. Lately, comparisons in the mechanisms of action between acupuncture and neuromodulation have received considerable attention [16,17]. Other literature suggests that acupuncture treatment is effective for PD symptoms, modulating inflammation and brain functional connectivity [15]. In this context, the development of acupuncture strategies based on shared mechanisms with neuromodulation, as personalized neuromodulating therapies, will provide new treatment options for patients with PD [16].

In previous studies of our group, we found evidence of the benefits of acupuncture in the quality of life of PD patients and promising results in reducing motor and non-motor symptoms [18]. Other studies have shown changes in brainstem auditory-evoked responses following electro-acupuncture therapy in chronic pain patients [19]. More specifically, acupuncture tended to improve hypo-metric gait, and rearranged activation of the cerebral cortex [12,20].

However, as far as we know, there is no evidence supporting the fact that acupuncture can improve the response time in PD patients. Considering the involvement of the deficit in reaction time in diminishing the quality of life of PD patients, in this study we aimed to evaluate (a) whether acupuncture can improve the motor time response after an auditory stimulus and (b) the differences between random and rhythmic inputs in the reaction time. 

## 2. Materials and Methods

This research focused on examining the effects of acupuncture treatment on RT in four PD patients [21]. Ethical approval for the study was obtained from the Ethics Committee of the Abel Salazar Institute of Biomedical Sciences at the University of Porto (reference no GS/HCC/9). Additionally, data collection was authorized by the CHUDSA/ICBAS committee, ensuring adherence to privacy regulations governing personal data handling. All collected data were handled confidentially, prioritizing the privacy and anonymity of participants to protect their information.

### 2.1. Participants

**Figure 1:**
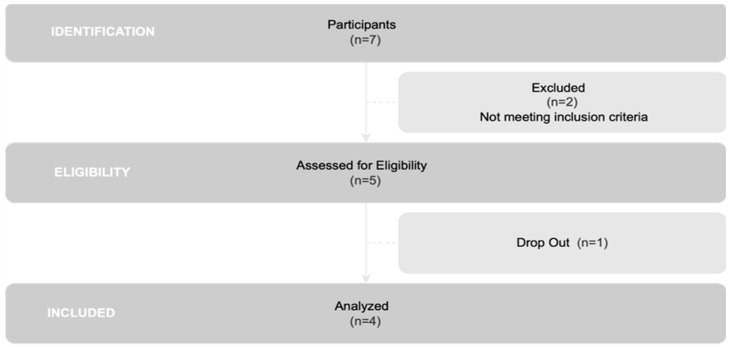
Flowchart of participant selection.

All members registered in the Associação Portuguesa de Doentes de Parkinson were invited to participate in this research. Initially, 7 individuals expressed interest and completed a questionnaire (n = 7). Following a thorough review of inclusion and exclusion criteria, 5 patients met the criteria for inclusion in the study (n = 5). However, one patient was unable to participate in the final evaluation, resulting in his withdrawal from the study. Consequently, the final analysis was performed on a group of 4 individuals (n = 4), as illustrated in Figure 1.

To qualify for inclusion in the study, participants needed to have a confirmed medical diagnosis of PD as assessed by a clinician [22,23], be classified within stages I–IV on the Hoehn and Yahr Scale [24], have a diagnosis aligning with the *shaoyin* stage according to the Heidelberg model of Traditional Chinese Medicine (TCM), maintain medical stability for at least 3 months, be new to acupuncture treatments, and provide a fully completed Informed Consent form [20]. 

To mitigate potential biases, we excluded individuals with needlestick phobia [25], dermal lesions at the acupuncture site, pregnancy or lactation, malignant tumors, bleeding disorders, or those undergoing anticoagulant therapy, psychiatric conditions [26], cognitive impairments hindering comprehension of instructions, and neuro-musculoskeletal alterations that could interfere with experimental procedures such as knee prostheses [27].

Furthermore, patients with other neurological conditions aside from PD, including idiopathic PD, primary PD, Parkinson-plus syndromes, and secondary Parkinsonism [28], as well as those with cardiac or renal insufficiency [29], and individuals undergoing DBS [30,31], were also excluded from the study.

In addition to these criteria, we established specific circumstances as dropout indicators, including (1) withdrawal of Informed Consent, (2) more than two unexplained absences from the intervention protocol, or (3) the occurrence of moderate-to-severe adverse effects. 

### 2.2. Materials

**Figure 2:**
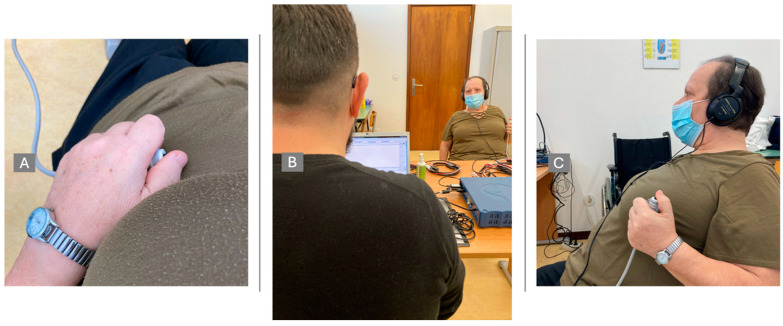
Evaluation procedures. (**A**)—Device to click, to evaluate motor response; (**B**)—Full-scenario data collection; (**C**)—Patient with auditory stimuli and motor response device.

For this study, we employed a Biopac MP36 polygraph with an associated digital headphone and a digital switch, both from Biopac as well, as shown in Figure 2, to evaluate alterations in reaction time.

Patients were asked to place the headphones and the button in one hand. Test always started on the right side (right hand) and was then repeated with the left side (left hand). This served to assess differences between hemispheres, and so, differences between the hemi corpus.

The patient was placed in a noise-free place and was asked to close his or her eyes to obtain all the attention required, without any distractions. The test consisted of two segments of sound stimuli: one with 10 random stimuli and another with 10 rhythmic stimuli, separated by 3 s each. The patient had 8 records: 2 random segments before treatment (right/left); 2 rhythmic segments before treatment (right/left); 2 random segments after treatment (right/left); and 2 rhythm segments after treatment (right/left).

The reaction time was given by the Biopac software (version 4.1) in milliseconds and the averages were calculated for comparison between the before and after treatment.

### 2.3. Procedures

Upon consenting to participate, patients were instructed to adhere to their prescribed medication regimen and clinical requirements. All patients had maintained stable medication doses for at least three months without experiencing any adverse effects.

**Figure 3:**
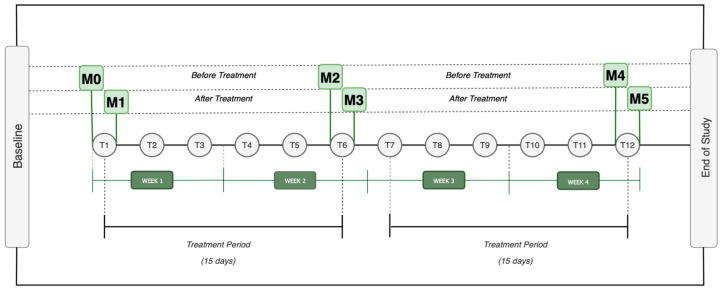
Design Study Legend: M0 = Moment zero; M1 = Moment one; M2 = Moment two; M3 = Moment three; M4 = Moment four; M5 = Moment five; T1 = Treatment one (…) T12 = Treatment 12.

During the study, every participant received three acupuncture sessions per week over a four-week period, amounting to a total of twelve treatments. The research plan consisted of six assessment-time moments, separated into three phases, as illustrated in Figure 3. Each phase involved two evaluation moments: one before and one after an acupuncture treatment, with a gap of at least 15 days between them. Thus, M0, M2, and M4 corresponded to evaluations conducted before acupuncture sessions, while M1, M3, and M5 represented assessments immediately following the treatment sessions.

### 2.4. Evaluation

Before and after each acupuncture treatment, the RTs were assessed using a digital acquisition system (Biopac Student Lab Pro—BSL 4.0 MP 36, Biopac Systems, Inc., Goleta, CA, USA).

The examiner made sure that the patient was listening to the auditory stimuli and tested if all the instructions were understood before starting the real test (Figure 2). 

Prior to beginning the acupuncture therapy, patients were provided with comprehensive instructions and explanations. During the treatment sessions, patients reclined in a peaceful, well-ventilated environment, ensuring their comfort throughout. They were recommended to wear loose attire that facilitated access to areas such as the knee, ankle, elbow, and wrist, and were encouraged to opt for shorts and sleeveless tops, accompanied by bare feet. Researchers maintained rigorous hygiene protocols, consistently washing their hands, and employing sterile surgical gloves for each patient, to ensure the utmost cleanliness and safety. 

A uniform acupuncture method, adhering to the principles of the Heidelberg Model of TCM, was applied, following the procedures detailed in a preliminary investigation [20], based on Greten [7,32,33,34].

**Figure 4:**
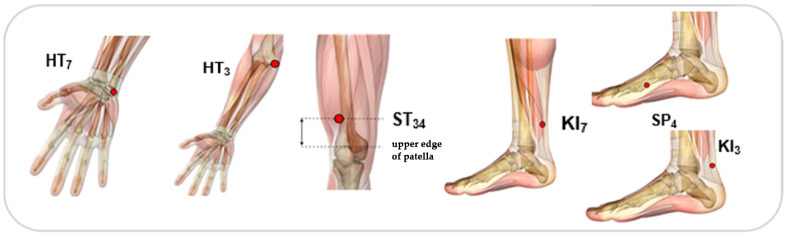
Acupoints used: ST_34_ (Liáng Qiu), HT_3_ (Shào Hăi), HT_7_ (Shén Mén), KI_3_ (Taì Xī), KI_7_ (Fù Liū) and SP_4_ (Gong Sūn).

Each participant received a total of 14 needles during each session, inserted bilaterally across the body and manually adjusted until a notable *DeQi* sensation was felt. These procedures were carried out by an acupuncturist with a master’s degree in TCM and three years of clinical practice. Sterile disposable needles with dimensions of 0.25 × 0.25 mm were employed. The acupuncture protocol focused on specific acupoints defined by the World Health Organization (WHO), as illustrated in Figure 4, and was administered 30 min after medication intake. Each treatment session lasted for 30 min [20]. All patients were taking the same drugs; however, they took different dosages, since they were at different stages of disease progression.

## 3. Results

Table 1 provides a summary of the clinical characterization of the four individuals included in the sample.

Table 2 outlines the response time obtained at each patient evaluation. Assessing how patients responded to treatments, according with their own individuality, was quite difficult. To address this difficulty, the division of patients into subgroups based on their pathological and biological characteristics—referred to as subtypes—has proven to be a promising approach for enhancing precision medicine. Recently, three subtypes of PD were reported [35]:

- Inching Pace subtype (PD-I), with mild baseline severity and mild progression speed.
- Moderate Pace subtype (PD-M), with mild baseline severity, but advancing at a moderate progression rate.
- Rapid Pace subtype (PD-R), with the most rapid symptom-progression rate.

According to these subgroups, patient 1 was classified as being PD-M subtype, and patients 2, 3 and 4, PD-I subtype (Table 2).

Every patient takes the same drugs, although with different dosages. Patient 1 takes 1 g, patients 2 and 3 take 800 mg and patient 4 takes 400 mg. In all of them, dyskinesias were observed. 

Importantly, no adverse effects were reported following any of the acupuncture treatments.

Using the data gathered from the 6 evaluation moments, four distinct outcomes were analyzed for each patient:

(A) Outcome A assessed the immediate effects of a single acupuncture session, comparing results before and after the intervention (M1-M0, M3-M2, and M5-M4).
(B) Outcome B investigated the accumulative effects of acupuncture pre-treatment, by comparing results obtained prior to two moments of treatments (M2-M0 and M4-M2).
(C) Outcome C examined the accumulative effects of acupuncture post-treatment, by comparing values obtained after two moments of treatments (M3-M1 and M5-M3).
(D) Outcome D permitted the analysis of the overall accumulative effects of the acupuncture protocol, by comparing the value obtained before the first treatment with the one obtained before the last evaluation moment (M4-M0).

### 3.1. Case Results

#### 3.1.1. Patient 1

**Figure 5:**
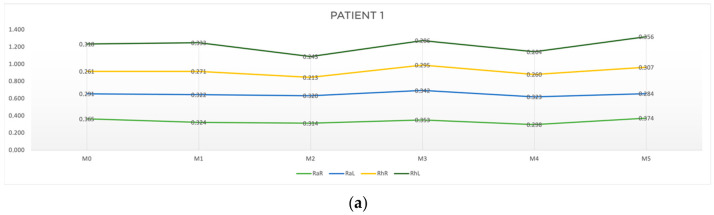

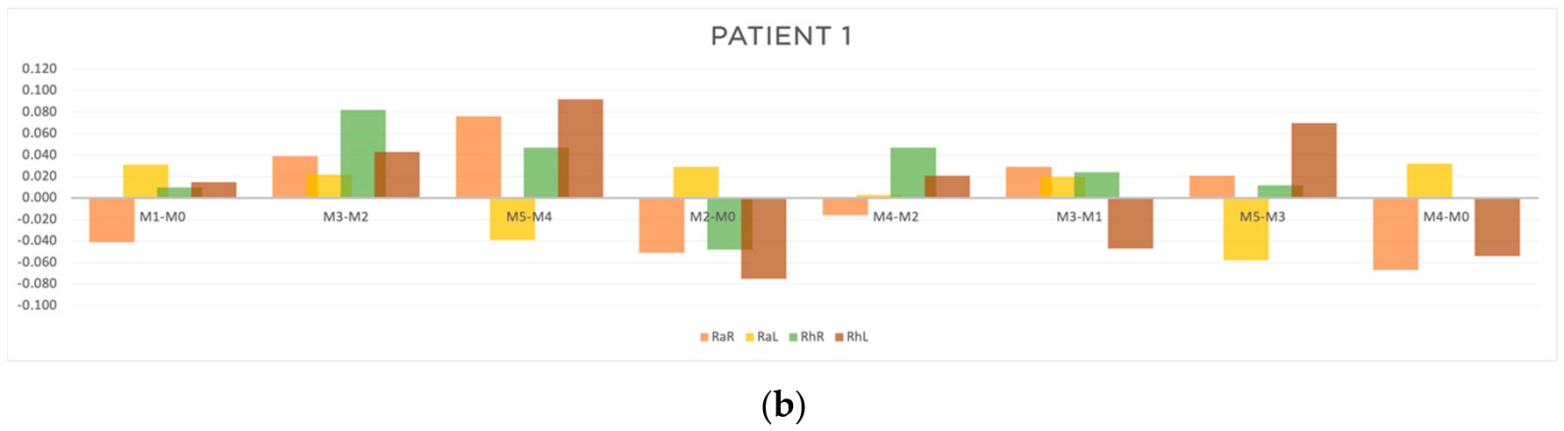
Graphic representation of (**a**) evolution in time and (**b**) differences between evaluation moments corresponding to A, B, C and D outcomes of patient 1. Legend: Random Right (RaR), Random Left (RaL), Rhythmic Right (RhR), Rhythmic Left (RhL). Legend: Random Right (RaR), Random Left (RaL), Rhythmic Right (RhR), Rhythmic Left (RhL).

Table 1 and Table 2 show, respectively, the characterization and the motor reaction-time results obtained for patient 1. Table 3 presents the results of the four outcomes analyzed for patient 1. Figure 5a) depicts the results obtained in the six moments of evaluation, reflecting the progression of the patient in terms of time response, and Figure 5b) shows, in a graphic way, the results for outcomes A, B, C and D of patient 1. 

As can be seen in Table 3 and Figure 5b, with respect to outcome A, a reduction in response time in the random collection was observed, namely at M1-M0 on the right side (RaR, t = −0.041 s) and at M5-M4 on the left side (RhL, t = −0.039 s). Concerning outcome B, there was an improvement for both random and rhythmic stimuli at M2-M0 (RaR, t = −0.051 s; RhR, t = −0.048 s; RhL, t = −0.075 s) and at M4-M2 (RaR, t = −0.016 s). Regarding outcome C, we found a cumulative improvement in time of response on the left side at M5-M3 (RaL, t = −0.058 s and at M3-M1 (RhL, t = −0.047 s). Finally, regarding outcome D, there was a reduction in response time both in the random and rhythmic component (RaR, t = −0.067 s; RhR, t = −0.001 s; RhL, t = −0.054 s).

The results of the patient 1 evaluation revealed the following:

- The acute effect (outcome A) of the treatment was more pronounced in the random component and on the right side (the more affected side).
- The cumulative component without treatment interference (outcome B) showed a reduction in response time, with a greater emphasis on the right side.
- The cumulative component with treatment interference (outcome C) had more expression on the left side.
- After one month (outcome D), the acupuncture protocol had positive results on both components, random and rhythmic, with more emphasis on the right side.
- Overall, the random responses showed a greater reduction in response time than rhythmic ones.

#### 3.1.2. Patient 2

**Figure 6:**
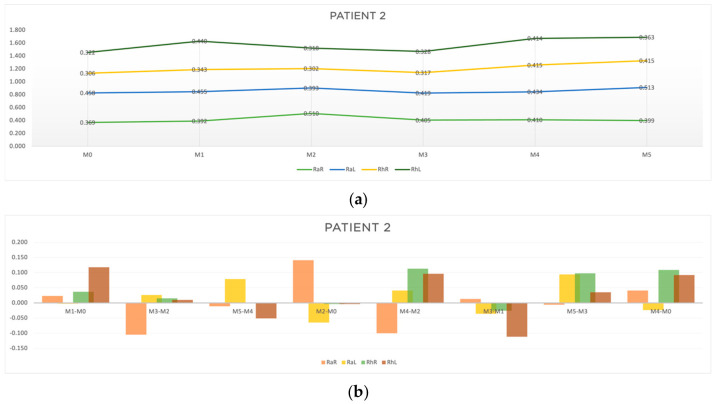
Graphic representation of (**a**) evolution in time and (**b**) differences between evaluation moments corresponding to A, B, C and D outcomes of patient 2. Legend: Random Right (RaR), Random Left (RaL), Rhythmic Right (RhR), Rhythmic Left (RhL). Legend: Random Right (RaR), Random Left (RaL), Rhythmic Right (RhR), Rhythmic Left (RhL).

The clinical characterization and response-time results at all the evaluation moments for patient 2 are shown in Table 1 and Table 2, respectively. Table 4 presents the results for the four outcomes analyzed. In Figure 6, the progression of patient 2 through the acupuncture treatment (a), and the results of the analyzed outcomes (b) are represented in a graphic way.

Regarding outcome A, the acute results had a greater expression in the random component on the right side at M3-M2 (RaR, t = −0.105 s) and at M5-M4 (RaR, t = −0.011 s). As for outcome B, improvements in response time were observed mainly for the random stimuli at M2-M0 (RaL, t = −0.065; RhR, t = −0.004; RhL, t = −0.004) and at M4-M2 (RaR, t = −0.10). Regarding outcome C, improvements in the rhythmic component at M3-M1 (RaL, t = −0.036; RhR, t = −0.026; RhL, t = −0.112) and M5-M3 (RaR, t = −0.006) were found. The results for outcome D showed improvements in the random movement on the left side (RaL, t = −0.024) (Table 4 and Figure 6b).

From the results obtained for patient 2, we can point out the following:

- The random responses showed a greater reduction in response time than the rhythmic ones.
- The cumulative effects of the acupuncture protocol (outcome B and D) seemed to be more relevant than the acute ones.
- The response to the overall protocol tended to be more pronounced for random stimulus on the left side (the more affected side).

#### 3.1.3. Patient 3

**Figure 7:**
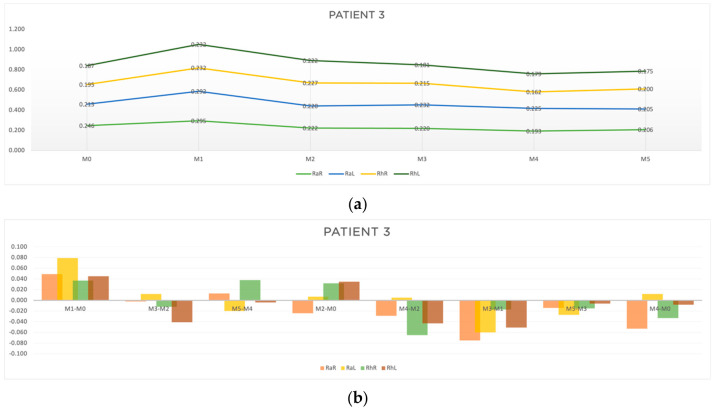
Graphic representation of (**a**) evolution in time (**b**) differences between evaluation moments corresponding to A, B, C and D outcomes of patient 3. Legend: Random Right (RaR), Random Left (RaL), Rhythmic Right (RhR), Rhythmic Left (RhL). Legend: Random Right (RaR), Random Left (RaL), Rhythmic Right (RhR), Rhythmic Left (RhL).

The clinical characterization and the response time at all the evaluation moments for patient 3 are shown in Table 1 and Table 2, respectively. The results for the four outcomes analyzed are presented in Table 5. In Figure 7, the progression of the patient through the treatment (a) and the results of the outcomes A, B, C and D (b) are represented in a graphic way. 

As can be seen in Table 5 and Figure 7b, the acute results (outcome A) were more pronounced at M3-M2 (RaR, t = −0.002 s; RhR, t = −0.012 s; RhL, t = −0.041), and at M5-M4 (RaL, t = −0.020 s; RhL, t = −0.004). It is worth noting that at M1-M0 there was no acute improvement in reaction time. As for outcome B, there was a considerable improvement in the right random responses at M2-M0 (RaR, t = −0.024 s) and at M4-M2 (RaR, t = −0.029 s) and in the left rhythmic response at M4-M2 (RhR, t = −0.065; RhL, t = −0.063). Outcome C showed improvements in response time for all the stimuli analyzed (Table 5 and Figure 7b). Outcome D showed a reduction in reaction time at M4-M0 in the random and rhythmic responses (RaR, t = −0.053 s; RhR, t = −0.033; RhL, t = −0.008 s).

Summarizing the results of patient 3:

- For rhythmic stimuli, we found a slightly greater reduction in response time when compared to random responses.
- The accumulative effects of acupuncture post treatment showed a reduction in response time for all the parameters analyzed.
- The overall response to the acupuncture protocol seemed to be more pronounced on the right side (the more affected side).

#### 3.1.4. Patient 4

**Figure 8:**
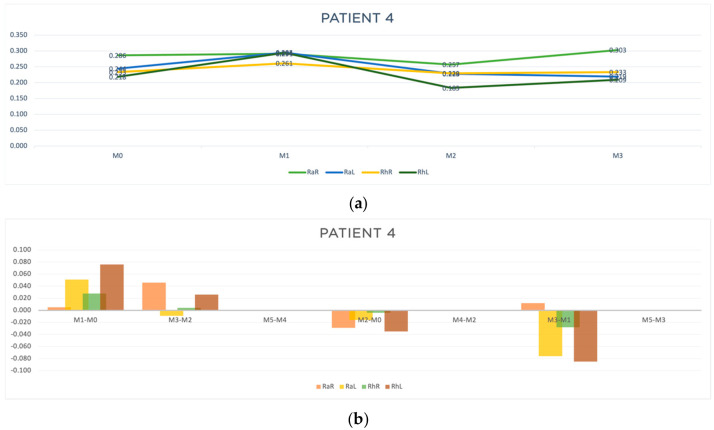
Graphic representation of (**a**) evolution in time (**b**) differences between evaluation moments corresponding to A, B and C outcomes of patient 4. Legend: Random Right (RaR), Random Left (RaL), Rhythmic Right (RhR), Rhythmic Left (RhL). Legend: Random Right (RaR), Random Left (RaL), Rhythmic Right (RhR), Rhythmic Left (RhL).

In Table 1 and Table 2 are shown, respectively, the clinical characterization and the results of the motor reaction time obtained for patient 4. Table 6 presents the results for the four outcomes analyzed. In Figure 8, the progression of the patient (a) and the results of the outcomes A, B, C and D (b) are represented in a graphic way. For this patient, M4 and M5 moments were not possible to evaluate, due to equipment damage.

Regarding patient 4, acute response improvement was observed only at M3-M2 (RaL, t = −0.009 s). However, regarding outcomes B and C, in the moments that we were able to evaluate, improved results were observed in almost all parameters (Table 6 and Figure 8b), indicating an accumulative effect of the acupuncture treatment in reducing the response time to random and rhythmic stimulus.

## 4. Discussion

Evaluation of motor symptoms in PD is based on clinical rating scales performed by clinicians. Thus, clinical neurological assessment of reaction time often involves subjective evaluations. To overcome this challenge and introduce a more quantitative and objective method, we conducted the present study using Biopac technology for RT analysis. Additionally, we used E-Rehabilitation technology, which was revealed to be adequate for clinical use due to its ability to quickly collect data and its portability [36].

The disruption in the motor circuit of the basal ganglia, caused by the loss of dopaminergic neurons in the nigrostriatal pathway observed in PD, contributes to the emergence of symptoms such as gait deficit. This gait impairment is characterized by reduced postural adjustments and postural instability, which typically results in falls. Postural instability has been correlated with slower information-processing speed, and so simple reaction-time tasks can be used to measure the speed of information processing [37]. 

Reaction time is defined as the time interval between a specific stimulus and the start of muscle response. We know that the level of neuronal network damage can be characterized by the reaction time in simple cognitive and motor tests [38], which means that poor performance in a simple reaction task can complement staging and evaluation in PD patients [37]. Different exploratory studies were conducted to explore the feasibility of reaction-time tests as predictors of falls, and positive results [11,39] were found. Nevertheless, there are some controversies about the existence and nature of reaction-time deficits in Parkinson’s disease, mainly because of the use of precures to speed movement (motor preprogramming) and the effects of medication on reaction time. But there is evidence in the literature that points to Parkinson’s disease as possibly causing a consistent deficit in simple reaction time [9]. 

Despite this controversy around the time of response in PD, some studies reported that patients with PD had similar mean choice stepping reaction times compared to healthy controls but presented significantly greater intra-individual variability [13]. However, other researchers observed overall increased response times in patients with Parkinson’s disease, compared to healthy controls [10]. Another study found that the PD group responses were significantly slower than the ones of controls, but auditory reaction times were significantly faster than visual [40]. 

Without a comparison between the responses of healthy individuals and Parkinson’s patients, our results are limited. However, they seem to suggest that, as the disease progresses, there is a tendency for an increase in motor response time to auditory stimuli, whether rhythmic or random, with a more pronounced decline in random stimuli. So, we can conclude that RT could be a sensitive tool for motor function assessment in PD patients. In this sense, auditory stimuli could facilitate a step initiation, conceivably by facilitating a stimulus identification process and increasing attentional control of stepping behavior, without influencing a decision-making process, even in a cognitively demanding condition, as in patients with PD [41].

Acupuncture is considered a safe and beneficial complementary approach in treating stroke [42], Parkinson’s disease, and various neurological disorders [43,44,45]. More specifically, it appears that acupuncture activates various brain amplitudes involved in pain regulation, processing emotions, cognition, and other brain regions [46]. Different studies found that acupuncture seems to protect dopaminergic neurons against toxic insults and increase dopamine production in the brain by inducing the release of neurotrophic factor, enhancing antioxidant agents, and inhibiting inflammatory processes [47]. Other researchers obtained evidence that acupuncture stimulates both innate and adaptive immune responses, with its anti-inflammatory effects involving the activation of neural reflexes [48]. In this sense, recent studies have revealed some organizational rules on how acupuncture stimulates autonomic somatosensory pathways. Activation of these pathways modulates various physiological functions in the body, such as systemic inflammation [49], contributing to restoring immune homeostasis [48,50]. Acupuncture also seems to regulate the balance between pro-inflammatory Tregs and anti-inflammatory Th17, as well as between pro-inflammatory Th1 and anti-inflammatory Th2. Maintaining the ratio of CD4+/CD8+ T cells, while controlling the quantity and activity of CD8+ T, acupuncture can contribute to maintaining the immune homeostasis of the body [50]. Results of different studies have shown that the therapeutic effect of acupuncture on nerve injuries focuses on the anti-oxidation pathway, neuroprotective processes, and anti-inflammatory effects. Lines of evidence indicate that the regulation of neuroendocrine and immune networks may be a common switch for acupuncture in different nervous system diseases [51]. With recent advances in physio pathological studies, there is now a great opportunity to gain insights into how acupuncture acts in modulating the nervous system [50].

Inflammation and microglial activation play crucial roles in PD and other neurodegenerative diseases that often present a chronic inflammatory response in the brain. This can be caused by neuronal damage, oxidative stress, or the presence of misfolded proteins, such as α-synuclein in PD. Chronic inflammation is mediated by the release of inflammatory cytokines, such as TNF-alpha, IL-1β, and IL-6. These molecules promote inflammation and may contribute to neuronal degeneration. In neurodegenerative diseases, microglia can become hyperactive or dysregulated. When pathologically activated, microglia cells release inflammatory substances that can exacerbate neuronal death. Thus, microglial activation can result in a vicious cycle, where inflammation caused by microglia damages nerve cells, leading to further microglial activation and additional inflammation. Inflammation and microglial activation also contribute to the progressive death of neurons, facilitating the accumulation of toxic proteins, such as α-synuclein, which forms neurotoxic aggregates and exacerbates neuronal degeneration. In Parkinson’s disease, this is particularly evident in brain areas where dopamine loss occurs, such as the substantia nigra. Therapeutic approaches aiming to reduce inflammation or regulate microglial activation are being explored as potential treatments for neurodegenerative diseases [52]

Although different studies validated the positive effects of acupuncture in PD symptoms, and thus support our findings, as far as we know, there are no previous works exploring the impact of acupuncture on reaction time in PD patients or even on other neurological diseases. So, we believe that this study represents an important contribution in the development of our knowledge in this area. 

Our sample consisted of one female and three men. This predominance of PD in men is in accordance with the literature. The lower prevalence of PD in females is not well understood but may be partially explained by sex differences in nigrostriatal circuitry and possible neuroprotective effects of estrogen [53].

The results of our data collection also showed that the acupuncture protocol used leads to positive outcomes in increasing the speed of motor response after a sound stimulus in Parkinson’s patients. These results seem to be more pronounced on the most affected side (it is known that all patients begin to demonstrate motor symptoms of the disease on one side, which, with disease progression, always remains the most affected side). Other studies, evaluating the reaction time, reported that it was longer in the more-affected side and in more-advanced PD stages. Frontal cognitive function, which is indicative of motor programming and movement regulation and perseveration, was also closely related with reaction time [39]. 

In this context, the results obtained indicated that a greater reaction time can be characteristic motor features of PD, and so this parameter could be used as a sensitive tool for motor function assessment in PD patients. 

Despite the small size of our sample, our results pointed out that the patient’s response to the acupuncture protocol seems to be better in early stages of the disease or in younger individuals. In this sense, epidemiological studies showed that PD incidence generally increases with age [3,54]. This can be important in understanding the condition’s natural history and suggests the need to adapt the dose of acupuncture treatment for the more advanced stages of the disease. Also, we observed a tendency to obtain better results for response time when considering the accumulative effects of the acupuncture protocol rather than the acute effects.

In accordance with this hypothesis are the results of the study of Lei et al. (2023) [18], which found that the acupuncture treatment for treating motor symptoms in PD patients may need to reach a certain dose to obtain the best therapeutic effect. Nevertheless, a certain tolerance can be developed with excessive acupuncture stimulation. This study reported that when acupuncture was used for more than 3 times a week, with a dose of acupuncture treatment < 60 times, the UPDRS-III score increased with the increase of acupuncture dose, but the score decreased if the dose continued to rise (*p* = 0.020).

To substantiate the results obtained in this study, we postulated the possible mechanisms of action of acupuncture on motor reaction time:

(a) Modulation of the Basal Ganglia: Acupuncture may influence the function of the basal ganglia, a brain region involved in motor control and directly affected in Parkinson’s disease. Stimulation of certain acupuncture points might help to regulate dopamine flow in this region, improving coordination and motor response.
(b) Increased Release of Neurotransmitters: Acupuncture has been associated with the release of neurotransmitters such as dopamine, serotonin, and endorphins, which can enhance neuronal communication. In Parkinson’s patients, this could improve motor signal transmission, reducing reaction time [55].
(c) Regulation of the Autonomic Nervous System: Acupuncture may regulate the balance between the sympathetic and parasympathetic nervous systems, which can influence the speed and efficiency of motor responses by reducing muscle tension and optimizing motor coordination [55,56].
(d) Neuroplasticity: Acupuncture might promote neuroplasticity, the brain’s ability to reorganize itself by forming new neural connections. This is particularly beneficial for patients with neurodegenerative diseases, helping to compensate for neuronal loss and improving motor reaction time.
(e) Improved Cerebral Blood Flow: Acupuncture can increase blood flow in specific brain areas involved in motor control. This increase in oxygen and nutrient supply may optimize neuronal function, resulting in a faster and more efficient motor response [52,57].

These hypothetical mechanisms suggest that acupuncture acts in a multifactorial manner, promoting both direct effects on the motor system and improvements in the neurological and biochemical environment, contributing to a reduction in motor reaction time. This theory could be developed in future studies.

Despite the limitations of the present study concerning the reduced sample size, and no comparison with controls and the limited outcomes analyzed, we consider that the results are promising and justify the continuity of the study, as our findings hint at the potential of acupuncture as a complementary approach for addressing the motor symptoms of PD patients. Further investigations are also required to clarify the clinical impact of the RT on the activity of daily living of patients with PD [39]. 

We believe that the next step should involve conducting a meticulously designed large-scale trial to validate and strengthen the observations and hypotheses raised here. Hence, the continuity of the study will be necessary to explore whether acupuncture can effectively ameliorate motor symptoms in PD. Consequently, acupuncture could be integrated as part of a multidisciplinary treatment approach for PD patients. Subsequent studies assessing other motor outcomes inherent to the process, such as nerve conduction velocity, electromyography of affected muscles, and functional magnetic resonance imaging studies, are of utmost importance for validating these findings [47]. We believe that those studies could also be an important contribution to increasing the accuracy of early diagnosis [58]. 

## 5. Conclusions

This investigation focused on assessing the clinical effects of acupuncture in the behavior of motor time response of PD patients. From the results obtained, we can conclude the following, overall:

(a) Our acupuncture protocol reduced the motor response time.
(b) The improvements in response time were more pronounced on the most affected side.
(c) Older patients or those at more advanced stages of the disease (III and IV) tended to present greater initial random-response values, compared to rhythmic values. On the other hand, in younger patients or patients at earlier stages of the disease, this difference is less noticeable. This can indicate that with the progression of the disease, there is a tendency for a decline in both rhythmic and random responses, with greater emphasis on the random one. However, the findings related to the differences between younger and older participants cannot be generalized, due to the small sample size.
(d) The timing/intensity of therapy should be adjusted, according to the stage of disease progression. More studies are necessary to determine the intervention dosage.

Although further investigations are needed, we can conclude that our preliminary findings pointed to the potential of acupuncture as a complementary approach for addressing the motor symptoms of PD patients, as well as the possible role of RT as a sensitive parameter that could be used as a tool for motor function assessment in PD patients.

## Figures and Tables

**Table 1:** Sample characterization.

	Variables	Age	Gender	Disease Degree	Affected Side	Subtype	Major Symptoms
Patients							
ID 01		72	Female	4	Right	PD-M	Gait and balance
ID 02		63	Male	3	Left	PD-I	Muscular Stiffness
ID 03		48	Male	2	Right	PD-I	Muscular Stiffness and Gait and Balance
ID 04		48	Male	1	Right	PD-I	Tremor

**Table 2:** Result of the motor reaction time in each patient and evaluation moment.

	Time		Random Right (RaR) Time t (s)	Random Left (RaL) Time t (s)	Rhythmic Right (RhR) Time t (s)	Rhythmic Left (RhL) Time t (s)
Patients						
P 1		M0	0.365	0.291	0.261	0.318
		M1	0.324	0.322	0.271	0.333
		M2	0.314	0.320	0.213	0.243
		M3	0.353	0.342	0.295	0.286
		M4	0.298	0.323	0.260	0.264
		M5	0.374	0.284	0.307	0.356
P 2		M0	0.369	0.458	0.306	0.322
		M1	0.392	0.455	0.343	0.440
		M2	0.510	0.393	0.302	0.318
		M3	0.405	0.419	0.317	0.328
		M4	0.410	0.434	0.415	0.414
		M5	0.399	0.513	0.415	0.363
P 3		M0	0.246	0.213	0.195	0.187
		M1	0.295	0.292	0.232	0.232
		M2	0.222	0.220	0.227	0.222
		M3	0.220	0.232	0.215	0.181
		M4	0.193	0.225	0.162	0.179
		M5	0.206	0.205	0.200	0.175
P 4		M0	0.286	0.244	0.233	0.218
		M1	0.291	0.295	0.261	0.294
		M2	0.257	0.228	0.229	0.183
		M3	0.303	0.219	0.233	0.209
		M4	*	*	*	*
		M5	*	*	*	*

Legend: Random Right (RaR), Random Left (RaL), Rhythmic Right (RhR), Rhythmic Left (RhL), *: Evaluation was not possible.

**Table 3:** Results of the effects of acupuncture in patient 1, based on the difference in response time at different evaluation moments. Outcomes: A— (M1-M0, M3-M2, M5-M4), B—(M2-M0), C— (M3-M1, M5-M3), and D—(M4-M0).

	Outcomes	Random Right (RaR) Time t (s)	Random Left (RaL) Time t (s)	Rhythmic Right (RhR) Time t (s)	Rhythmic Left (RhL) Time t (s)
Evaluation Moments					
M1-M0		−0.041	0.031	0.010	0.015
M3-M2		0.039	0.022	0.082	0.043
M5-M4		0.076	−0.039	0.047	0.092
M2-M0		−0.051	0.029	−0.048	−0.075
M4-M2		−0.016	0.003	0.047	0.021
M3-M1		0.029	0.020	0.024	−0.047
M5-M3		0.021	−0.058	0.012	0.070
M4-M0		−0.067	0.032	−0.001	−0.054

Legend: Random Right (RaR), Random Left (RaL), Rhythmic Right (RhR), Rhythmic Left (RhL).

**Table 4:** Results of the effects of acupuncture in patient 2, based on the difference in response time at different evaluation moments. Outcomes: A— (M1-M0, M3-M2, M5-M4), B—(M2-M0), C— (M3-M1, M5-M3), and D—(M4-M0).

	Outcomes	Random Right (RaR) Time t (s)	Random Left (RaL) Time t (s)	Rhythmic Right (RhR) Time t (s)	Rhythmic Left (RhL) Time t (s)
Evaluation Moments					
M1-M0		0.023	−0.003	0.037	0.118
M3-M2		−0.105	0.026	0.015	0.010
M5-M4		−0.011	0.079	0.000	−0.051
M2-M0		0.141	−0.065	−0.004	−0.004
M4-M2		−0.100	0.041	0.113	0.096
M3-M1		0.013	−0.036	−0.026	−0.112
M5-M3		−0.006	0.094	0.098	0.035
M4-M0		0.041	−0.024	0.109	0.092

Legend: Random Right (RaR), Random Left (RaL), Rhythmic Right (RhR), Rhythmic Left (RhL).

**Table 5:** Results of the effects of acupuncture in patient 3, based on the difference in time response at different evaluation moments. Outcomes: A— (M1-M0, M3-M2, M5-M4), B—(M2-M0), C— (M3-M1, M5-M3), and D—(M4-M0).

	Outcomes	Random Right (RaR) Time t (s)	Random Left (RaL) Time t (s)	Rhythmic Right (RhR) Time t (s)	Rhythmic Left (RhL) Time t (s)
Evaluation Moments					
M1-M0		0.049	0.079	0.037	0.045
M3-M2		−0.002	0.012	−0.012	−0.041
M5-M4		0.013	−0.020	0.038	−0.004
M2-M0		−0.024	0.007	0.032	0.035
M4-M2		−0.029	0.005	−0.065	−0.043
M3-M1		−0.075	−0.060	−0.017	−0.051
M5-M3		−0.014	−0.027	−0.015	−0.006
M4-M0		−0.053	0.012	−0.033	−0.008

Legend: Random Right (RaR), Random Left (RaL), Rhythmic Right (RhR), Rhythmic Left (RhL).

**Table 6:** Results of the effects of acupuncture in patient 4, based on the difference in time response at different evaluation moments. Outcomes: A— (M1-M0, M3-M2, M5-M4), B—(M2-M0), C— (M3-M1, M5-M3), and D—(M4-M0).

	Outcomes	Random Right (RaR) Time t (s)	Random Left (RaL) Time t (s)	Rhythmic Right (RhR) Time t (s)	Rhythmic Left (RhL) Time t (s)
Evaluation Moments					
M1-M0		0.005	0.051	0.028	0.076
M3-M2		0.046	−0.009	0.004	0.026
M5-M4		*	*	*	*
M2-M0		−0.029	−0.016	−0.004	−0.035
M4-M2		*	*	*	*
M3-M1		0.012	−0.076	−0.028	−0.085
M5-M3		*	*	*	*
M4-M0		*	*	*	*

Legend: Random Right (RaR), Random Left (RaL), Rhythmic Right (RhR), Rhythmic Left (RhL), *: Evaluation was not possible.

## Data Availability

The raw data supporting the conclusions of this article will be made available by the authors on request.
